# Treatment of coronal knee angular deformities in children by a modified métaizeau percutaneous transphyseal screw technique

**DOI:** 10.1007/s00264-025-06695-x

**Published:** 2025-12-02

**Authors:** Karim Abdallah, Alhassan M. Abdelhamid, Ahmed M. Bashendi, Ahmed Samir Barakt, Hazem Abd El-Hameed, Mohamed M. Hegazy, Mohamed Tageldeen Mohamed

**Affiliations:** 1 Orthopedic surgery and traumatology department, Kasr Al-Ainy Faculty of Medicine, Cairo University, Egypt, Cairo, Egypt; 2 El Helal Hospital, Cairo, Egypt, Cairo, Egypt; 3 Orthopedic surgery department, University of Medical Sciences & Technology, Khartoum, Sudan, Khartoum, Sudan

**Keywords:** PETS, Genu varum, Genu valgum, Coronal angular deformities of the knee

## Abstract

**Purpose:**

Knee coronal angular deformities are a frequently encountered challenge in paediatric orthopaedic practice. When surgical treatment is indicated, guided growth techniques have many advantages in managing these conditions. The purpose of this study is to evaluate the outcome of a modification of the original Percutaneous Epiphysiodesis using Transphyseal Screw (PETS) technique described by Métaizeau as a minimally invasive surgical approach in the treatment of knee angular deformities.

**Methods:**

In this prospective study, a total of 14 patients (comprising 25 limbs) with a coronal plane deformity of the knee underwent percutaneous transphyseal screw hemiepiphysiodesis. Operative time is assessed. The patients were subsequently monitored for an average duration of 28 months.

The radiological assessment was conducted using the metrics of MAD (mechanical axis deviation), mLDFA (mechanical lateral distal femoral angle), and MPTA (medial proximal tibial angle). Clinical assessment included the intermalleolar distance (IMD) and intercondylar distance (ICD). The functional outcome evaluation was conducted using a modified version of the original Böstman score, taking into account the different age groups of the targeted cases.

**Results:**

In the genu valgum group, the mean preoperative values were: intermalleolar distance (IMD) 16.9 cm, mechanical axis deviation (MAD) 2.6 cm, and mechanical lateral distal femoral angle (mLDFA) 84°.

In the genu varum group, the mean preoperative values were: intercondylar distance (ICD) 8.4 cm, mechanical axis deviation (MAD) –3.0 cm, and medial proximal tibial angle (MPTA) 77.8°.

The mean operative time was 15 min. All radiological and clinical outcome measures showed significant improvement (P ≤ 0.05). At 24 months, 96% of cases achieved an excellent Böstman knee score. One patient reached skeletal maturity before full correction could be achieved. No other complications were observed.

**Conclusion:**

This modification of the Métaizeau technique retains the advantages of PETS and offers a simplified approach that may reduce operative time and fluoroscopy use. Our results suggest that it is a safe and effective option for correcting coronal angular knee deformities in children. Further comparative studies are needed to confirm these potential benefits.

## Introduction

Coronal knee angular deformities are common in childhood [1]. Angular deformity can be caused by a wide range of conditions, varying from physiological variations to different pathological conditions. A proper and thorough patient evaluation is vital for successful differential diagnosis [2].

Knee malalignment causes differentially high stresses on the medial or lateral compartment of the knee joint, increasing the likelihood of accelerated degenerative processes [3]. In developing children, they produce cosmetic issues (especially if severe or bilaterally asymmetrical), gait difficulties (especially in patients with genu valgum who might compensate by axially rotating their limbs to clear out their knees), and worsening activity-related aches [4]. Timely interference to treat knee angular deformities is needed to avoid effects such as altered gait patterns, early articular cartilage degeneration, and soft tissue changes [5, 6].

Angular malalignment treatment includes restoring normal mechanical axis alignment and joint orientation lines through corrective osteotomy or guided growth [7, 8]. Guided growth has established its place as an effective treatment option for coronal knee deformities. Different techniques and modifications have been described, with every new modification avoiding the previous ones' shortcomings and adding new advantages [9].

PETS was introduced by Métaizeau in 1998 [10]. It is considered the least invasive procedure of all growth plate manipulation techniques, with the most cosmetically appealing scars. The growth modulation achieved with the PETS technique occurs rapidly after implantation and can be completed by removing the screw once full correction is obtained [11].

In this study, we assess the results of a modification of the Métaizeau transphyseal screw technique first described by Shah et al. [12] that theoretically could avoid some of the difficulties some surgeons may encounter during guide wire aiming and placement. The purpose of this prospective case series was to evaluate the feasibility, reliability, and reproducibility of this modified percutaneous transphyseal screw in children as a minimally invasive guided growth surgical intervention for the treatment of knee coronal plane deformity.

## Materials and methods

### Study design

Following the permission of the ethics committee, we proceeded to perform a prospective case series study. This study included patients diagnosed with coronal plane deformity of the knee who underwent percutaneous transphyseal screws and met the eligibility criteria for enrollment.

### Participants

This study was undertaken to investigate a cohort of 14 patients (comprising 25 affected limbs). Before enrollment, the parents or caregivers signed an informed consent after receiving detailed information about the operation, alternative procedures, and possible complications.

Inclusion criteria were skeletally immature patients with angular knee deformities, the anatomical location of the deformity at the distal femur and the proximal tibia, a physis with a satisfactory degree of residual growth of approximately two years till maturity, patients with intermalleolar distance (IMD) exceeding 10 cm for the genu valgum group, and the intercondylar distance (ICD) surpassing 6 cm for the varum group.

Exclusion criteria encompass patients whose expected growth remaining duration is less than 12 months, the presence of an abnormal or a closed physis, the presence of a fixed flexion deformity exceeding 15 degrees, genu recurvatum, patients with collateral ligament instability, presence of subluxation, and skeletal dysplasia.

A thorough preoperative assessment was conducted and documented. Before the surgical procedure, all patients had a standing radiograph of both lower limbs. The MAD, LDFA, MPTA, and JLCA were measured. The difference between the final follow-up measurement before screw removal and the preoperative measurement was calculated and then divided by the number of months between radiographs to assess the degree and rate of correction. Patients were followed three, six, twelve, eighteen, and twenty-four months after surgery. Any complications, such as pain, hemarthrosis, or failure of angular correction, were documented. CT was done after the 24-month treatment period to ensure the absence of a physeal bar at the site of the transphyseal screw.

Assessment of the functional outcome was done by using the Böstman knee score [13]. The Böstman knee score was modified, and school resumption was used instead of return to work because of the different age group targeted by this study.

### Surgical technique

To appreciate the difference between the original technique described by Métaizeau and the modification, we will briefly describe both techniques.

#### The original technique of Métaizeau

An incision was made over the distal femoral metaphysis or proximal tibial metaphysis. Then, under fluoroscopic guidance, a guidewire starting at the metaphysis was aimed to cross the physis and stop just short of the articular surface. More specifically, the wire was aimed to cross the physis at the center in the sagittal plane and the outer or inner quadrant in the coronal plane for genu varum or valgum, respectively. The guidewire was used to guide a cannulated drill bit. Then, a 7.3 mm screw was inserted so that at least four threads were placed in the epiphysis. All wounds were closed in layers, and a dry sterile dressing was applied [10].

#### The modified technique

All procedures were performed under general anesthesia with the administration of preoperative antibiotics. The patient was positioned in the supine posture. The guidewire insertion was modified to **a retrograde approach** for the distal femur and **an antegrade approach** for the proximal tibia. The entry point was selected at either the medial or lateral distal femoral condyle or tibial plateau, based on the deformity present. The modification made guidewire aiming and placement within the optimal physeal crossing point easier and less demanding than the original technique because the starting point is closer to the physis. This modification theoretically could decrease operative time by avoiding multiple guidewire placement trials. It may also decrease radiation exposure compared to the original technique.

Guidewire placement was confirmed using fluoroscopy, with both anteroposterior (AP) and lateral radiographs. In the AP view, the wire was positioned to pass through the medial or lateral quadrant of the physis, based on the deformity. In the lateral view, the wire was placed to pass through the center of the physis. The guide wire was advanced to exit the contralateral aspect of the thigh or leg and then withdrawn from the opposite end.

A 1 cm longitudinal skin incision was made along the wire trajectory, followed by blunt dissection of the fascia and muscle layers to expose the bone. The bone was drilled using a cannulated drill bit, stopping just short of the articular surface. A depth gauge was used to measure the screw length.

**Fig. 1:**
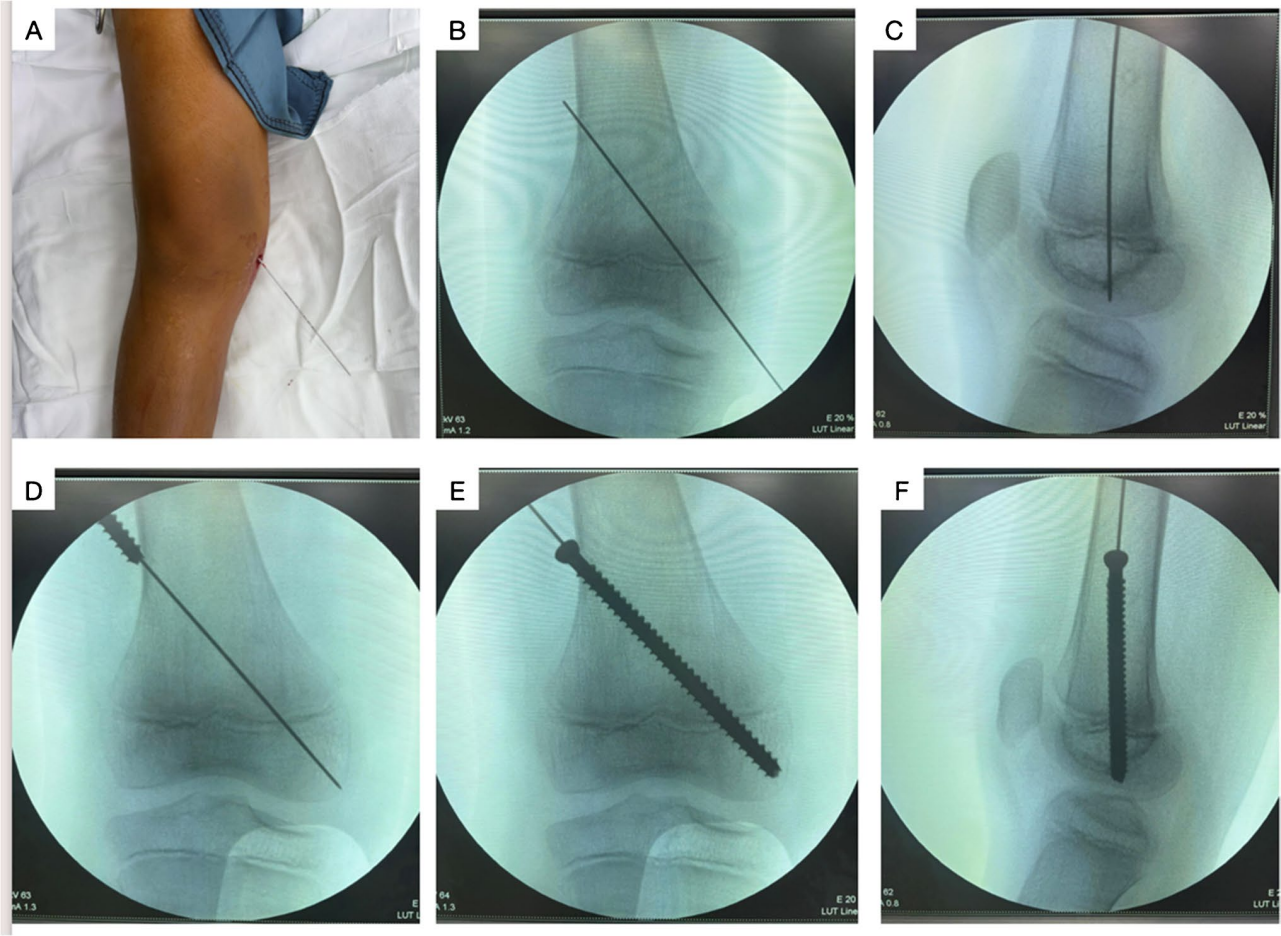
Steps of the modified PETS technique in a case of distal femoral genu valgum

A 6.5 mm cannulated fully threaded screw was inserted over the guidewire (antegrade in the distal femur and retrograde in the proximal tibia) with a minimum of four threads engaging beyond the physis (Fig. 1). Screw placement was confirmed on both AP and lateral fluoroscopic views. Alternatively, in patients younger than 7 years of age, we used a 4.5 mm cannulated fully threaded screws given the smaller bone profile. All wounds were closed in layers, and a dry sterile dressing was applied.

### Postoperative protocol

Analgesia was given post-operatively as needed. Full weight-bearing was allowed as soon as tolerated. When patients were able to ambulate, they were discharged and told to avoid sports for two weeks.

Follow-up was done after two weeks for wound check-ups, and at regular three-month intervals thereafter to monitor the rate of correction. Outcome was measured according to the clinical improvements, radiographic measurements, and functional assessment. Screws were removed after appropriate correction of angular deformity was achieved.

### Statistics

Statistical Package for Social Sciences (SPSS) computer program (version 19 windows) was used for data analysis. P value ≤ 0.05 was considered significant. Results are expressed as minimum, maximum, median, mean and standard deviation or number (%). Test of normality, Kolmogorov–Smirnov test, was used to measure the distribution of data measured pre-operation. Data was found not normally distributed, so comparison between variables measured at pre- and post-operation within the same group was performed using Wilcoxon Sign Ranks test.

## Results

14 patients (comprising 25 limbs) were included in this study. Nine males and five females. The mean age was 8.21 years. There were 19 limbs with distal femoral genu valgum and six with proximal tibial genu varum. The mean operative time was 15 min, and the mean follow-up was 28 months (Table 1).

**Table 1:** Patients’ demographics

Mean age	8.21 years (range, 4–12)
Males: females	9:5 (64:36%)
Type of deformity	Valgum 19 (76%) Varum 6 (24%)
Side affected	Unilateral: 3 Bilateral: 11
Side affected according to type of deformity	Valgum 1 Unilateral: 9 Bilateral Varum 2 Unilateral: 2 Bilateral
Mean operative time for single limb (from skin incision to dressing application)	15 min (range, 11–18)
Mean follow-up period	28 months (range, 24–36)

Radiological and clinical correction occurred in 23 of 25 limbs (92%). The duration of correction refers to the time between surgery and implant removal. In the valgum subgroup, mean IMD showed statistically significant improvement from 16.88 cm to 2.19 cm postoperatively. In the varum subgroup, mean ICD also significantly improved from 8.4 cm to 1 cm postoperatively. All radiographic parameters showed significant improvement, as shown in Tables 2 and 3.

**Table 2:** Relevant descriptive statistics for distal femoral genu valgum limbs (*n* = 19)

	Minimum	Maximum	Mean	SD	*P*-value
Pre IMD (cm)	14	20	16.88	1.75	0.001
Post IMD (cm)	2.0	2.5	2.19	0.25	
Pre MAD (cm)	3.0	6.0	4.58	0.96	0.001
Post MAD (cm)	0.0	1.0	0.79	0.98	
Pre mLDFA (°)	74	83	78.74	3.02	0.001
Post mLDFA (°)	85	91	88	3.56	
Correction time (months)	6	13	10.63	2.06

**Table 3:** Relevant descriptive statistics for proximal tibial genu varum limbs (*n* = 6)

	Minimum	Maximum	Mean	SD	*P*-value
Pre ICD (cm)	6	10	8.4	1.67	0.04
Post ICD (cm)	0.5	2.5	1	0.42	
Pre MAD (cm)	−2.0	−6.0	−3.0	0.0	0.02
Post MAD (cm)	−1.0	0.0	−0.17	0.41	
Pre MPTA (°)	75	81	77.8	2.39	0.04
Post MPTA (°)	88	92	90.2	1.48	
Correction time (months)	12	15	13.5	1.05	

One 11-year-old female patient with bilateral genu valgum had reached skeletal maturity before full correction, requiring distal femoral metaphyseal osteotomy. No wound complications were recorded. CT was done 24 months postoperatively to ensure the absence of physeal bar, none developed any at the site of the transphyseal screw.

We utilized the Böstman knee score at 24 months postoperatively to assess the functional results of this percutaneous technique. The total score fell into excellent (30–28 points), good (27–20 points), and unsatisfactory (< 20 points) [26]. For the genu valgum cases, 18 limbs showed an excellent outcome, while one had a good outcome. All six limbs with genu varum attained excellent outcomes (Table 4).

**Table 4:** Functional outcome according to Böstman knee score (using our suggested modification for the selected age group)

	Valgum (*n* = 19)	Varum (*n* = 6)	Total (*n* = 25)
Good	1 (5.26%)	0 (0.0%)	1 (4.0%)
Excellent	18 (94.74%)	6 (100.0%)	24 (96.0%)

## Discussion

Correction of angular deformities around the knee using guided growth is considered the gold standard treatment for skeletally immature patients with coronal angular knee deformities. Different guided growth procedures have evolved with time. These include the Phemister permanent epiphyseodesis technique, Blount’s staple, percutaneously guided grooved staple, hemiepiphysiodesis using a transphyseal screw, and insertion of a plate and screws [14, 15]. Each new procedure was introduced to address the disadvantages of previous techniques.

Blount and Clarke introduced staples for the correction of angular deformities in 1947, and their technique has been adopted for decades [14]. A rapid and distractive physeal growth environment caused most of the staples to bend, break, or migrate. The use of multiple staples failed to address these issues consistently. Furthermore, because of growth, most staples got embedded deep into the bone, and their removal was frequently difficult, resulting in local bone loss that could jeopardize the reversibility of this technique. This casts doubt on the wisdom and necessity of using such rigid implants, and their popularity has decreased as a result [14].

Dissatisfied with some of the previously stated stapling issues, the concept of using an extra-periosteal, non-locking plate as a tension band (TBP) was introduced by Stevens [15]. With this method, the growth modification effect takes some time until the implant causes physeal tethering and compression. This may cause undercorrection in patients approaching skeletal maturity. Other issues noticed were postoperative pain, broken screws, rebound, superficial surgical site infection, and the scars left behind by the wounds used to implant and extract them. [16].

PETS is a minimally invasive technique popularized by Metaizeau that has proven its efficacy in the treatment of angular knee deformities and limb-length discrepancy [10]. Advantages include smaller, cosmetically appealing scars and a rapid growth modification following implantation. Removal of PETS after deformity correction can be technically challenging and often requires a larger incision compared to the initial procedure. Kim et al. conducted a comparative study of 33 patients (90 limbs) with idiopathic genu valgum, evaluating outcomes of percutaneous epiphysiodesis using transphyseal screws (PETS) in 90 limbs against tension-band plating (TBP) in 60 limbs. Both techniques achieved reliable correction of the deformity; however, the rate of correction was significantly faster in the PETS group. In the distal femur, the mean correction rate was 0.92° per month with PETS compared to 0.64° per month with TBP, while in the proximal tibia, the rates were 0.72° per month and 0.55° per month, respectively [17].

If we take the distal femur into perspective, the original Metaizeau technique involved an antegrade guidewire and an antegrade screw direction starting from the metaphysis towards the epiphysis. Abdelaziz et al. have proposed a modification of this technique involving a retrograde guidewire-retrograde screw; their technique showed effective deformity correction [18]. In our study, we utilized a modification of a retrograde guidewire- antegrade screw, which was described by Shah et al. and Chand et al. as technical notes [12, 19]. We hypothesized that a retrograde guidewire placement is technically easier as the starting point is closer to the physis, minimizing angular translation of the guidewire at the physeal crossing point with changes in insertion trajectory, allowing easy placement of the guidewire with the least number of trials. This may save operative time and decrease radiation exposure.

Metaphyseal screw head placement in the antegrade femoral technique is safer than its placement at the articular epiphyseal end in the retrograde screw; the latter may cause painful ROM, stiffness, difficult removal, hemarthrosis, septic arthritis, and possible articular cartilage damage. In the study by Abdelaziz et al., seven patients suffered from painful knee flexion [18]. Although they didn’t report any other complications than pain, intraarticular hardware placement may be associated with potential morbidity, and it could happen upon implementing their technique with other hands.

We believe that the technique utilized in this study combines the advantages of both the original Metaizeau and Abdelaziz techniques.

A major concern with PETS in general was the fear of a permanent growth arrest caused by the trans-physeal screw, but several studies have shown that this technique is reversible and that growth resumption usually proceeds normally following screw removal [10, 18]. In this study, A CT performed at 24-month follow-up failed to demonstrate the presence of any physeal bar at the site of trans-physeal screw following its removal.

We have not reported any cases of infection. There were no cases with genu recurvatum detected on follow-up, presumably because the screws were all inserted in the central part of the sagittal plane. No complications or difficulties were noted during screws removal. One patient, an 11-year-old female with bilateral genu valgum, had reached skeletal maturity before full correction and required distal femoral metaphyseal osteotomy.

An important consideration in the application of guided growth techniques is their variable efficacy in older children, particularly girls above ten years of age. As physeal growth potential diminishes with age, the corrective effect of guided growth becomes less predictable, and the results in this subgroup may be equivocal. This limitation should be kept in mind when counseling families regarding expected outcomes.

In patients approaching skeletal maturity, we believe PETS would be beneficial given its rapid onset of correction compared to other guided growth techniques. We still consider guided growth worthwhile in this group, as even partial correction can reduce the extent and potential morbidity of any subsequent osteotomy needed to complete the correction. Moreover, PETS remains a harmless, minimally invasive, well-tolerated procedure.

We believe that this is the first study in the literature describing the effectiveness of this modified PETS technique with a mean follow-up of 28 months. Study limitations include a lack of a control group and the small number of cases, which may have decreased the power for statistical analysis. We recommend future comparative studies and randomized controlled trials studying different PETS techniques to provide stronger evidence.

This modification of the Métaizeau technique retains the advantages of PETS and offers a simplified approach that may reduce operative time and fluoroscopy use. Our results suggest that it is a safe and effective option for correcting coronal angular knee deformities in children.

## Data Availability

No datasets were generated or analysed during the current study.
